# Applied models and molecular characteristics of small cell lung cancer

**DOI:** 10.3389/pore.2024.1611743

**Published:** 2024-04-22

**Authors:** Gabriella Mihalekné Fűr, Kolos Nemes, Éva Magó, Alexandra Á. Benő, Petronella Topolcsányi, Judit Moldvay, Lőrinc S. Pongor

**Affiliations:** ^1^ Cancer Genomics and Epigenetics Core Group, Hungarian Centre of Excellence for Molecular Medicine (HCEMM), Szeged, Hungary; ^2^ Genome Integrity and DNA Repair Core Group, Hungarian Centre of Excellence for Molecular Medicine (HCEMM), Szeged, Hungary; ^3^ Department of Pulmonology, Szeged University Szent-Gyorgyi Albert Medical School, Szeged, Hungary; ^4^ 1st Department of Pulmonology, National Koranyi Institute of Pulmonology, Budapest, Hungary

**Keywords:** SCLC, drug response, ctDNA, liquid biopsy, databases

## Abstract

Small cell lung cancer (SCLC) is a highly aggressive type of cancer frequently diagnosed with metastatic spread, rendering it surgically unresectable for the majority of patients. Although initial responses to platinum-based therapies are often observed, SCLC invariably relapses within months, frequently developing drug-resistance ultimately contributing to short overall survival rates. Recently, SCLC research aimed to elucidate the dynamic changes in the genetic and epigenetic landscape. These have revealed distinct subtypes of SCLC, each characterized by unique molecular signatures. The recent understanding of the molecular heterogeneity of SCLC has opened up potential avenues for precision medicine, enabling the development of targeted therapeutic strategies. In this review, we delve into the applied models and computational approaches that have been instrumental in the identification of promising drug candidates. We also explore the emerging molecular diagnostic tools that hold the potential to transform clinical practice and patient care.

## Introduction

Small Cell Lung Cancer (SCLC) is an extremely aggressive form of cancer, accounting for about 15% of all lung cancer cases. Due to its aggressive nature, over 60% of SCLC cases already show metastasis at the time of diagnosis, despite regular imaging [1]. Consequently, surgical resection is rarely an option, leaving chemotherapy, radiation, and in some instances, immunotherapy, as the main treatment methods. This situation adversely affects SCLC research and the development of new molecular diagnostic tools as well, as tumor samples are rarely available. Since no major improvements have been achieved in SCLC treatment in over three decades, which is paired with short life expectancy, the National Cancer Institute to categorizes this disease as a “recalcitrant” cancer. Therefore, there is an urgent need for a more profound understanding of SCLC’s development and progression, the creation of more accurate models, and the development of new molecular diagnostic tools that can overcome the challenges presented by this complex disease.

## Subtypes of SCLC

A decade ago, SCLC was predominantly viewed as a uniform type of pulmonary neuroendocrine cancer. The World Health Organization (WHO) and the National Comprehensive Cancer Network (NCCN) still classify SCLC into two subtypes: small cell carcinoma (previously known as oat cell carcinoma) and combined-SCLC, characterized by features of both small and non-small cell carcinoma [2].^1^ When SCLC cell lines were first developed approximately 30 years ago, they revealed two distinct morphological subtypes: classic and variant subtypes. Classic cell lines formed non-adherent aggregates or spheroid cells, while variant cell lines exhibited either loosely adhering aggregates or formed tightly adhering monolayers [3].

**FIGURE 1:**
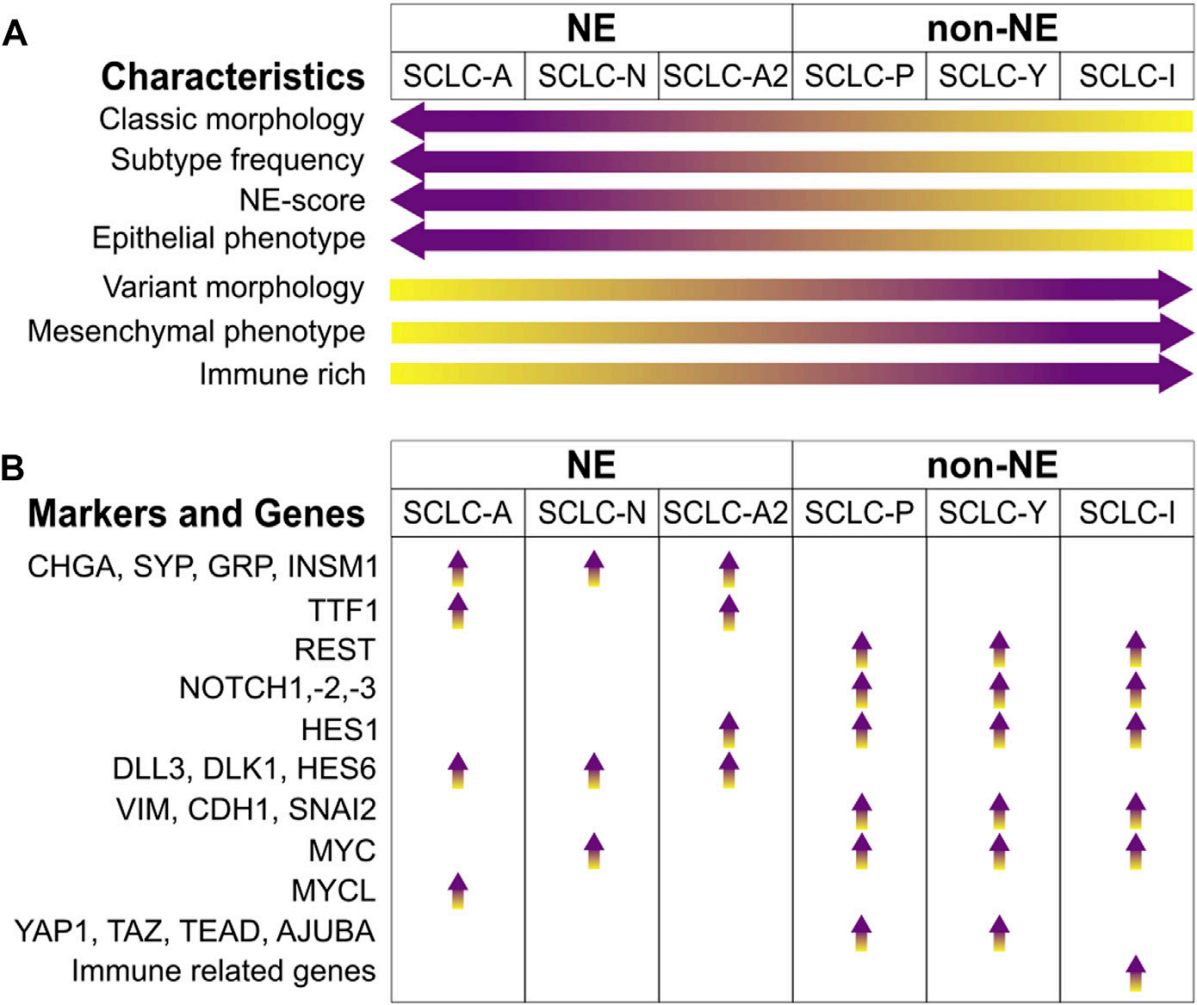
SCLC subtypes. **(A)** Signature enrichment of subtypes. **(B)** Key markers and genes enriched in the different SCLC subtypes. Abbreviations: CHGA, Chromogranin A; SYP, Synaptophysin; GRP, Gastrin-Releasing Peptide; INSM1, Insulinoma-Associated Protein 1; TTF1, Thyroid-Transcription Factor 1; REST, RE1-Silencing Transcription Factor; NOTCH1,-2,-3, Neurogenic Locus Notch Homolog Protein 1, -2, -3; HES1, Hes Family BHLH Transcription Factor 1; DLL3, Delta Like Canonical Notch Ligand 3; DLK1, Protein Delta Homologue 1; HES6, Hes Family BHLH Transcription Factor 6; VIM, Vimentin; CDH1, Cadherin 1; SNAI2, Snail Family Transcriptional Repressor 2; MYC, MYC Proto-Oncogene, BHLH Transcription Factor; MYCL, MYCL Proto-Oncogene, BHLH Transcription Factor; YAP1, Yes1 Associated Transcriptional Regulator; TAZ, Transcriptional Coactivator With A PDZ-Binding Domain; TEAD, TEA Domain Transcription Factors; AJUBA, LIM Domain-Containing Protein Ajuba.

The homogeneity of SCLC is exhausted by the prevalent TP53 and RB1 inactivation [4–7], from which new characterizations have been developed over the years. A critical finding was that SCLCs could be categorized based on their neuroendocrine (NE) characteristics, into NE (with high NE scores) and non-NE (with low NE scores) types by IHC staining for neuroendocrine markers such as SYP (Synaptophysin) or CHGA (Chromogranin A) [8]. Transcriptomic profiling of these cell lines has led to the identification of further subtypes based on the expression of transcription factors, a classification also supported by tumor sample analysis [7, 9–11], which we summarized in Figure 1.

The most prevalent subtype is characterized by elevated expression of Achaete-scute homologue 1 (ASCL1), termed SCLC-A, which is crucial in regulating neuroendocrine differentiation [12–14]. NEUROD1 (Neuronal Differentiation 1), another marker for the NE subtype, often co-exists with ASCL1. NEUROD1, enriched in the SCLC-N subtype, also influences NE differentiation and contributes to the progression of cancer, [14]. A less common group, which is negative for both ASCL1 and NEUROD1, falls into the non-NE category. These tumors and cell lines sometimes express ASCL2, suggesting its role as an alternative transcription driver [12]. The main non-NE subtypes are distinguished by the expression of POU2F3 (SCLC-P) and YAP1 (SCLC-Y). POU2F3 (POU Class 2 Homeobox 3), a key transcription factor in chemosensory tuft cells, is expressed in SCLC variants that share a similar expression profile with these cells, indicating a possible origin from this cell lineage [15]. YAP1 (Yes1 Associated Transcriptional Regulator) and TAZ (Transcriptional Coactivator with a PDZ-Binding Domain) are involved in the Hippo pathway as transcriptional coactivators and effector proteins, leading to tissue overgrowth and oncogenesis [16]. SCLCs expressing YAP1 represent a relatively rare subgroup [7, 11, 17].

The (NE) and non-NE subtypes of SCLC show distinct transcriptional signatures. Zhang et al. identified a set of 50 genes that are differentially expressed in SCLC tumors, cell lines, and genetically engineered mouse models (GEMMs) [12]. This set includes 25 genes closely associated with NE SCLCs and another 25 linked with non-NE SCLCs. These genes were used to generate an NE scoring system to help patient stratification. An ASCL1 subset that expresses HES1 (Hes Family BHLH Transcription Factor 1) was also identified and was termed as SCLC-A2 or NEv2 [18–20], which was often associated to liver metastases [19].

The CHGA and SYP genes are widely recognized NE markers, showing high expression levels in both SCLC-A and SCLC-N subtypes [9, 12]. Insulinoma-Associated Protein 1 (INSM1), a zinc-finger transcription factor found in developing neuroendocrine tissues [21], is indicative of SCLC and associated with NE characteristics [22, 23]. Additional genes linked with NE subtypes include Gastrin-Releasing Peptide (GRP) [7], Protein Delta Homologue 1 (DLK1) [24], and BEX1 (Brain Expressed, X-Linked 1) [25]. NKX2-1, the gene for Thyroid-Transcription Factor 1 (TTF1), is a transcriptional target of ASCL1, making its expression specific to the SCLC-A subtype [9, 26].

Notch signaling is known to facilitate a transition from NE to a chemoresistant non-NE phenotype in SCLC [20]. It activates the expression of REST (RE1-Silencing Transcription Factor), which suppresses the expression of NE markers such as ASCL1, SYP, or CHGA. Similarly, YAP1 has been found to support this Notch-induced shift to a non-NE phenotype [27]. Additionally, HES1 (Hes Family BHLH Transcription Factor 1) has a negative correlation with NE scoring [12], as its expression is governed by NOTCH1, which hinders the transcription of REST and YAP1 [27, 28]. While Notch signaling fosters non-NE differentiation, some genes, such as DLL3, DLK1 and HES6, which activate this pathway, have been found to correlate with the NE subtype, thereby acting in a pro-tumorigenic manner towards NE cells [12, 19, 29, 30].

Genes in the TGFβ pathway have been found to be expressed in non-NE cases of SCLC, acting as suppressors of ASCL1 [12, 19]. There is a crosstalk between the Notch and the Hippo pathway [31, 32]. YAP1 and TAZ are overexpressed in SCLC-Y, as well as the TEAD genes (TEAD2 and TEAD3) and AJUBA (Ajuba LIM Protein), negative regulators of the pathway [12, 19]. Additionally, the transition from NE to non-NE phenotype can be influenced by Notch and TGFβ signaling, which is linked with the process of epithelial to mesenchymal transition (EMT). EMT is known to promote metastasis and resistance to treatment in cancer cells [33]. The expression of the intermediate filament vimentin (VIM), and SNAI2 (Snail Family Transcriptional Repressor 2), a repressor of E-cadherin (CDH1), has been observed to negatively correlate with the NE state of SCLC [12, 20]. The MYC proto-oncogene paralogues (BHLH Transcription Factors), are differentially expressed in SCLC. MYCL being the target of ASCL1 is expressed in SCLC-A [19], while MYC, a target of NEUROD1, which drives to a non-NE phenotype is elevated in SCLC-N [34, 35] and in non-NE SCLC-Y [7] subtypes.

DNA replication stress is a key biological feature of SCLC [36]. The near universal loss of p53 and RB1 tumor suppressors is one reason of replication stress as they play roles in cell cycle progression [37]. Another reason could be the overexpression of MYC family of oncogenes, which promote heightened replication initiation, which lead to defects in replication [38, 39]. Replication stress is higher in NE tumors, presenting a specific gene expression pattern with genes to deal with the elevated replication rates, to hinder the DNA damage, DNA repair, and cell cycle related genes [19, 40]. The elevated replication stress observed in NE tumors may be a reason why many tumors have exceptional initial response [36].

A new subtype of small cell lung cancer (SCLC) has been recently identified, which does not fully align with the previous four subtypes based on transcription factor expression, although SCLC-Y is commonly found. Instead, this subtype, termed SCLC-inflamed or SCLC-I, is characterized by the expression of immune-related genes. SCLC-I tumors exhibit the highest levels of CD8^+^ T-cell and overall immune infiltration among all the subtypes. Additionally, SCLC-I is marked by high levels of immune checkpoint molecules (such as PD-L1, PDCD1, CTLA4, CD38, IDO1, TIGIT, VISTA, ICOS, LAG3), T cell attractant chemokines (CCL5, CXCL10), and MHC genes (HLA-DRB1, HLA-DQA1, MICA). This suggests they may be more responsive to checkpoint inhibitors compared to other subtypes. Gay et al., in a reanalysis of the *IMpower133* data, noted that SCLC-I tumors tend to respond favorably to carboplatin/etoposide/atezolizumab treatment [9, 41].

This highlights the heterogeneity of SCLC and underscores the importance of adopting these proposed subtype classifications. Continued subtype-specific research is essential to understand their distinct pathophysiologies and to identify optimal treatment strategies.

## Experimental models of SCLC

**FIGURE 2:**
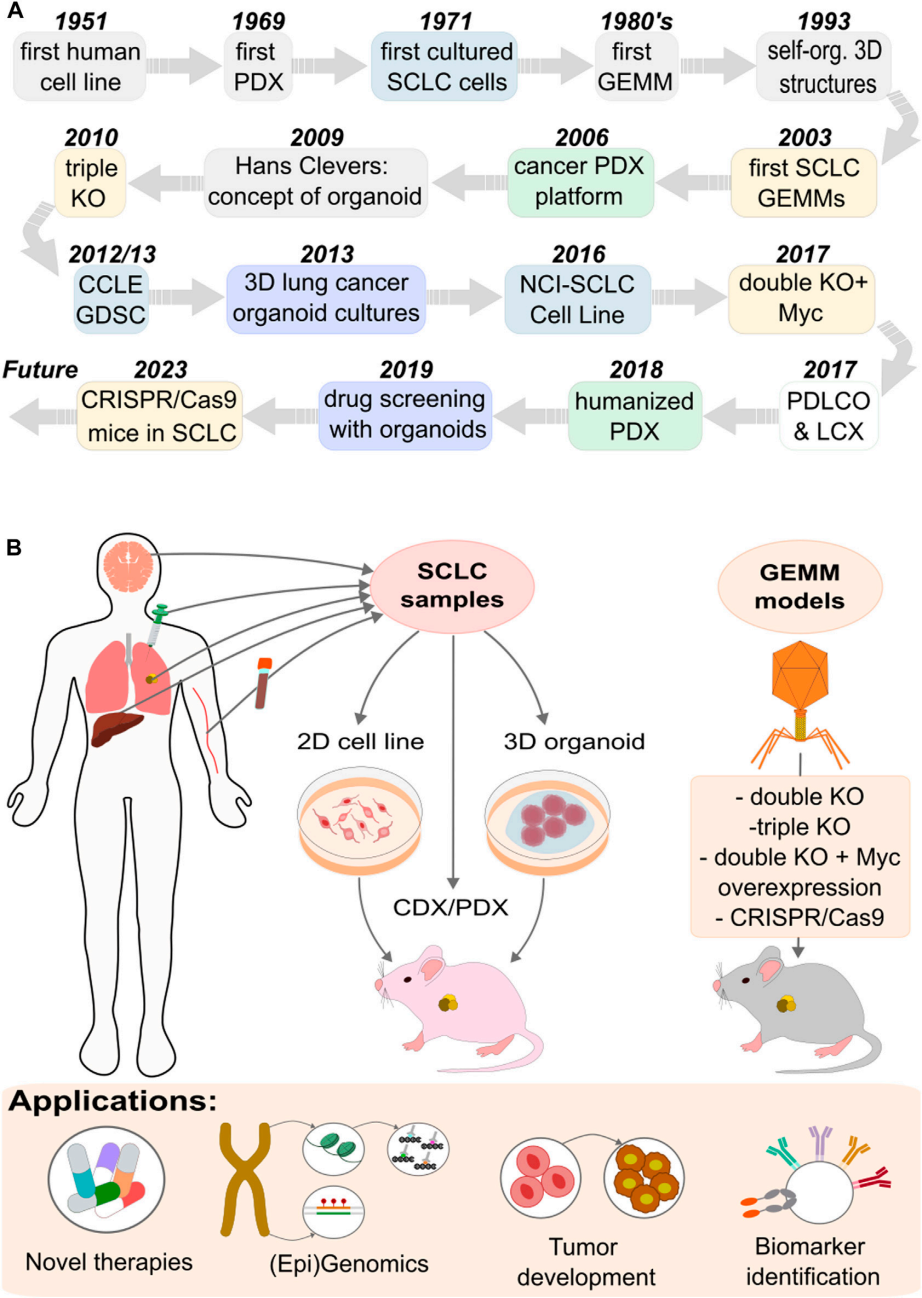
Experimental model systems used to explore SCLC. **(A)** Timeline of the developed models. **(B)** Approaches and applications of the different models.

There are several model systems used to examine cancer and SCLC (Figure 2). Each system has advantages and disadvantages, covering a wide range of application, such as drug testing, performing omics studies, or to characterize SCLC development (Table 1).

**TABLE 1:** Summary of advantages and disadvantages of each model.

Characteristics	SCLC models			
	Cell line	CDX/PDX	PDO	GEMM
Cost	**−**	**+**	**+**	**+**
Time consuming	**−**	**+**	**∙**	**+**
Difficult to generate	**−**	**+**	**∙**	**+**
Rapid expansion	**+**	**−**	**+**	**−**
Reproducibility	**+**	**−**	**−**	**∙**
Tumor heterogenity	**−**	**+**	**+**	**∙**
Original tumor biology	**∙**	**∙**	**+**	**+**
Primary disease	**+**	**∙**	**+**	**+**
Metastasis	**∙**	**+**	**+**	**∙**
Biomarker discovery	**∙**	**+**	**+**	**∙**
Drugscreening	**+**	**+**	**+**	**+**
Translational research	**−**	**+**	**+**	**∙**

### Patient derived cell lines (PDC)

The basic growth properties of SCLC were first defined using panels of cell lines developed from 1971 through the early 1990s [42–45] (Figure 2A). Primarily derived from metastatic SCLC tumors, these cell lines have been instrumental models for understanding gene functions and testing potential drug candidates.

During the early 1990s, the National Cancer Institute (NCI, Bethesda, MD), pioneered a new method for drug screening focused on specific diseases. This method involved using a collection of 60 human cancer cell lines from nine different cancer types [46, 47]. Originally, SCLC was not part of the NCI-60, which made drug predictions for this cancer type unfeasible. However, the advent of high-throughput technologies and improvements in characterizing cell lines have led to the development of more extensive cell line collections that now include SCLC. These databases contain detailed information on gene mutations, structural alterations, and changes in copy numbers, as well as mRNA expression profiles. This allows for comparative analyses across different cell lines and cancer types. For example, the Cancer Cell Line Encyclopedia (CCLE) [48] resource utilized data from massively parallel sequencing and microarray expression profiles from 947 human cancer cell lines, alongside the responses to 24 anticancer drugs in 479 of these lines. Additionally, the Genomics of Drug Sensitivity in Cancer (GDSC) has become a major public source for data on cancer cell drug sensitivity and molecular indicators of drug response [49]. The GDSC database includes information from nearly 75,000 experiments, covering responses to 138 anticancer drugs across approximately 700 cancer cell lines. Recent studies enable the exploration of the role of microRNAs as potential biomarkers in SCLC. By the early 1990s, investigations had already been conducted on 126 SCLC cell lines, providing insights into the response of these cell lines to anticancer drugs, and a library of investigational agents complemented by exon and microRNA arrays [50].

Despite their affordability and suitability for high throughput screening, these cells do not fully capture the complex nature of the tumor environment [51]. For this reason, such cancer models have roughly a 10% success rate in advancing anti-cancer drugs to clinical trial stages [52]. In addition, even the promising drug candidates usually failed at preventing recurrence in pre-clinical and clinical trials [52]. Nonetheless, they are still useful model organisms that can be used to better characterize and study what genetic and epigenetic factors affect SCLC growth and development, providing quick and easy tools for drug and CRISPR based screens.

### Patient derived organoids (PDO)

In 2009, Hans Clevers laid the foundation for organoid research, demonstrating new methods for organoid culture [53, 54] (Figure 2A), significantly boosting the development of patient-derived organoids (PDOs). The first lung cancer PDOs were generated by Inoue and coworkers [55, 56]. Compared to traditional cancer cell lines and patient-derived xenograft (PDX) models, lung cancer PDOs offer several advantages [57, 58]. PDO is a 3D structure culture formed from enriched patient cancer cells. It exhibits genetic stability, self-renewal capabilities, drug sensitivity, and high degrees of similarity to human organs in both structure and function [59].

A key attribute of PDOs is their faithful retention of the parental tumor’s genomic changes, yet they allow for faster modeling and some degree of gene editing [57, 60–62]. Through whole-exome sequencing, whole-genome sequencing, and RNA-seq Kim and their colleagues found that short-term cultured lung organoids retained 92.7% and 77% of the driver mutations found in the primary tissue, respectively [63, 64]. They developed 80 lung cancer organoid lines, including five from SCLC. These SCLC organoids accurately reproduced the tissue structure of the original tumors and maintained key SCLC diagnostic markers such as CD56, SYP, and TTF-1. It was also noted that the culture conditions for non-small cell lung cancer (NSCLC) PDOs and SCLC PDOs differed, with R-spondin1 and Wnt3a being crucial for the long-term culture of SCLC tumor organoids [57, 65]. In addition, Zhang et al. were able to establish 3D co-culture models to expand circulating tumor cells (CTCs) *ex vivo* from early-stage SCLC patients [66, 67].

Recent studies using engineered mouse lung cancer organoids (LCOs) have shed light on SCLC metastasis mechanisms, showing that KMT2C deficiency leads to extensive metastasis [68, 69]. This particular SCLC model, driven by Trp53 and Rb1 (mouse homologs of human TP53 and RB1, respectively) deficiencies and Myc overexpression, displayed multiple diagnostic markers of SCLC and developed significant distal metastases in multiple organs [68]. In SCLC research, brain organoids can be propagated on a large scale, facilitating the testing of various cell subtype combinations [70]. However, creating a PDO model is time-intensive, costly, and technically challenging, necessitating further research into PDOs [54].

### Patient and circulating tumor cell xenografts (PDX/CDX)

The *in vivo* preclinical methods of SCLC research include the application of mouse xenograft models. These models are either cell line-derived xenografts, created from SCLC cell lines, or PDX models, which involve directly implanting tumor material into immunocompromised mice like NOD/SCID or NSG [45, 71] (Figure 2B). The advantage of these models can be seen in the example of BH3 mimetics (BCL2/BCLxL inhibitors), where significant effectiveness in SCLC cell line models has been observed [45, 72, 73], with limited sensitivity in SCLC PDX models [74]. The discrepancy between PDX models and cell-line models in drug sensitivity underscores the potential impact of *in vitro* selection artifacts on clinical outcomes, suggesting that PDX models may better reflect the expression profiles and drug sensitivities of SCLC patient tumors [75, 76].

Obtaining SCLC samples is unfortunately very challenging as it is rarely surgically removed, and invasive tumor sampling is typically unnecessary after diagnosis [45]. To bridge this gap CTCs from the blood of cancer patients can be sampled noninvasively and are highly abundant in SCLC patients [77, 78]. In the CDX technique, changes in CTC numbers are closely aligned with chemotherapy responses, indicating that CTCs may reflect the biology of SCLC tumors. Both PDX and CDX techniques maintain the original human tumor’s histopathological and genetic characteristics, preserving its heterogeneity and complexity [45, 79]. This significantly improves the ability to identify and test biomarkers for treatment and prognosis [64].

PDX models are thought to preserve the tumor microenvironment and epigenetic features, which are crucial for tumorigenesis, invasion, metastasis, and the effectiveness of anticancer therapies [60]. However, PDX has several limitations, including chances of tumor tissue engraftment failure, a long tumor development timeline, dissimilarity of the tumor microenvironment between human and murine models, and low throughput for drug screening [80]. Furthermore, the requirement for immunocompromised hosts limits their use in studying cancer-immunity interactions. Advances in humanized mice and mice with reconstituted human immune systems offer potential solutions [54]. Sequencing studies using next-generation sequencing on SCLC PDX models have proven valuable for unraveling the molecular landscape of this disease [78], and also reported the presence of a concordant somatic TP53 mutation in all CTCs [77].

### Genetically engineered mouse models (GEMM)

Research in lung cancer has progressed significantly due to studies using genetically engineered mouse models (GEMMs), which facilitate the examination of tumor biology and environmental interactions *in vivo* conditions [78]. The first SCLC GEMM model was developed in the laboratory of Anton Bern in 2003 [10]. It incorporated loss-of-function mutations in the Rb1 and Trp53 tumor suppressor genes (double knockout), mirroring mutations found in over 90% of SCLC patients [81] (Figure 2A). These mutations were believed to exhibit significant histological and molecular biological similarities to the human disease, though the rate of tumor development in the model was slower than typically observed in human cases [82].

GEMMs laid the groundwork for numerous subsequent studies, which explored the roles of various potential tumor suppressors and oncogenes in SCLC [83]. A notable study within this framework examined P130, also known as Rbl2 (RB Transcriptional Corepressor Like 2), a member of the retinoblastoma (Rb) family (Figure 2B). The deletion of the P130 gene in mice already having Rb1 and Trp53 gene knockouts led to faster tumor growth, thereby affirming P130's tumor suppressor function in SCLC (triple knockout) [81, 84]. Similar methods were used to demonstrate the importance of PTEN and NOTCH tumor suppressors [7, 85].

Conditional Trp53/Rb1 (double knockout) and Trp53/Rb1/Rbl2 (triple knockout) knockout mouse models displayed traits typical of the ASCL1-high/NEUROD1-low subtype of human SCLC [35]. The double knockout model was further developed by introducing a CRE-activated Myc-T58A mutation. The stabilization of MYC in this model hastened tumor development and growth processes, and the resulting invasive tumors were representative of the high NEUROD1 subtype [35, 82].

Since the 2010s, CRISPR/Cas9 GEMMs have been applied in cancer research [86, 87], and significant breakthroughs occurred in 2023. These innovations include the development of mice capable of CRISPR/Cas9 base editing [88] and prime editing [89]. Prime editing, which employs Cas9 with a reverse transcriptase, allows for precise and efficient mutation engineering, as demonstrated in a proof-of-principle study introducing hotspot mutations for Kras and Trp53 in the lung and pancreas [90].

## Databases and tools to mine SCLC

Research on SCLC is guided by a wealth of data from specialized websites, analyzed using integrative approaches. This combination of resources is not only changing the paradigm of the disease but also improving its treatment. The scientific progress is centered around data repositories that provide raw genomic, proteomic, and clinical data, as well as analytical tools that interpret this data to derive meaningful disease insights.

### Websites for data in SCLC research

**FIGURE 3:**
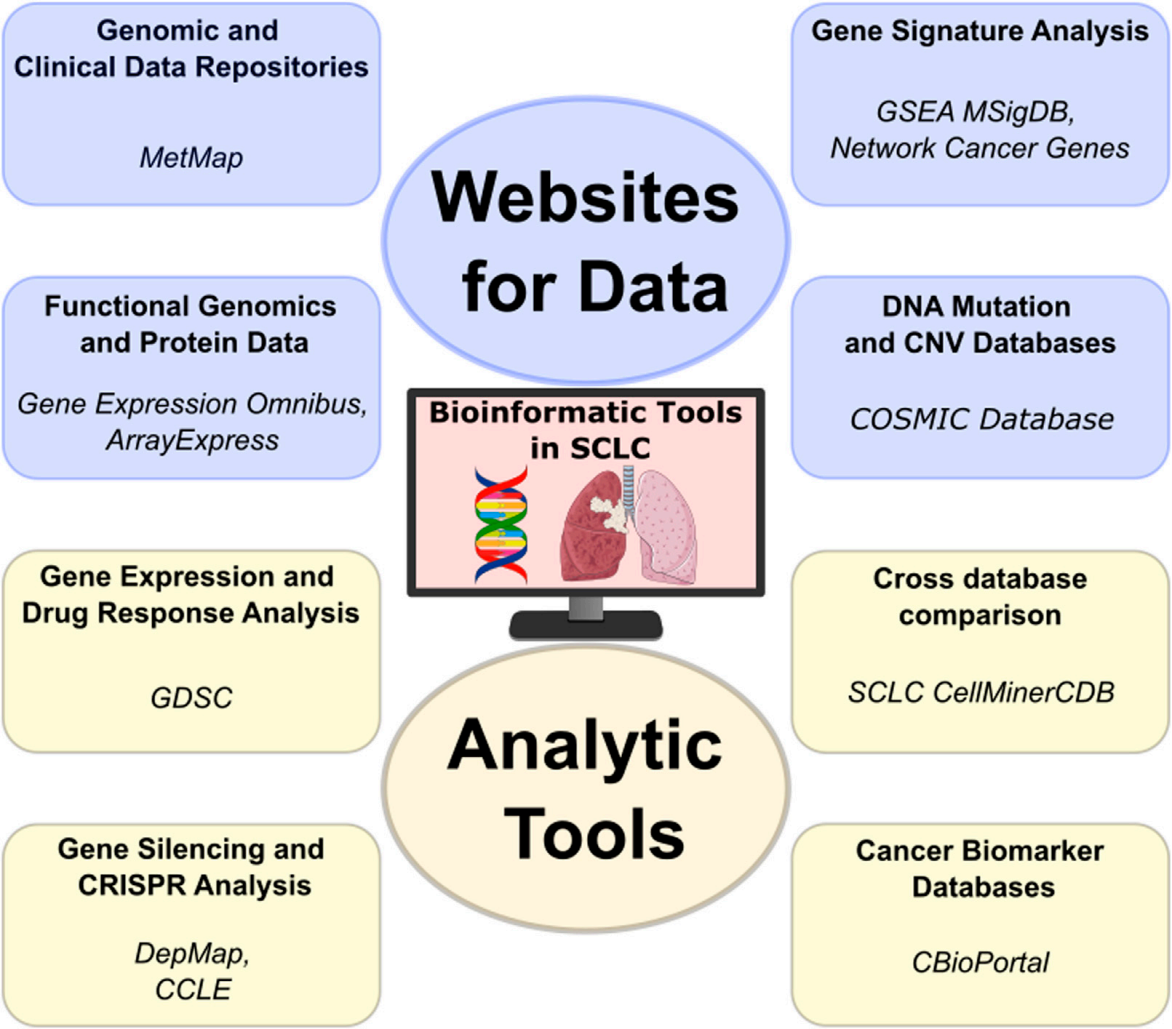
The analytic tools used in SCLC research are pivotal not only for their analytical capabilities but also for the downloadable databases they offer. This feature allows for versatile data manipulation, enabling researchers to conduct customized analyses that can lead to novel insights into SCLC’s molecular intricacies and potential treatments. (Lung—lung-cancer icon by Servier https://smart.servier.com/ is licensed under CC-BY 3.0 Unported https://creativecommons.org/licenses/by/3.0/, DNA—dna-nucleotides-ribbon icon by Servier https://smart.servier.com/ is licensed under CC-BY 3.0 Unported https://creativecommons.org/licenses/by/3.0/.

Data repositories have become indispensable in SCLC research (Figure 3). They serve not only as collections of genomic and clinical data but also as platforms that enable intricate comparative studies and groundbreaking translational research. Data can be directly accessed through different sites, such as the SRA (Sequence Read Archive) [91] and ENA (European Nucleotide Archive) [92] in case of experimental models, or European Genome-phenome Archive (EGA) [93] and dbGAP [94] for protected patient data that requires access. These allow researchers to process and analyze data in any preferred way. In many cases, processed data is also made available, such as at the GEO or ArrayExpress, which allows users to directly access results without having to reprocess large data amounts. Recent studies leveraging published data have revealed novel gene signatures that are correlated with SCLC progression [95–97]. Overall, these databases play a crucial role in discovering biomarkers, which aid in developing new diagnostic and prognostic tools. This, in turn, helps personalize patient care for SCLC.

Mutation databases such as Catalogue Of Somatic Mutations In Cancer (COSMIC) [98] offer a comprehensive record of genomic aberrations discovered in cancer, including SCLC. COSMIC documents genetic alterations, such as mutations and copy number variations (CNVs), which can be used to find recurrent alterations and hotspots through interactive plots. It is an essential resource for identifying genetic alterations in cancer, including SCLC, and for the continuous search for targeted therapies. In addition, the MetMap [99] provides a detailed profiling of metastatic potential of cell lines including SCLC, aiding in the identification of potential therapeutic targets.

Gene signature analysis, facilitated by platforms such as GSEA’s [100] MSigDB [101], and Network Cancer Genes [102], enables the identification of predictive gene patterns [103]. This approach is essential for developing targeted and personalized therapies for SCLC, tailoring treatments to individual genetic profiles [104].

### Analytic tools facilitating SCLC research

The analytical tools utilized in research on SCLC are crucial not only for their capabilities but also for the provided easy data access (Figure 3). This feature allows for versatile data manipulation, enabling researchers to conduct customized analyses that can lead to novel insights into SCLC’s molecular intricacies and potential treatments.

The Genomics of Drug Sensitivity in Cancer (GDSC) [49] offers a vast repository of data specifically focused on drug response in cancer, providing insights into how various cancer cells react to different treatments. Using the GDSC portal, users can compare drug sensitivity across cell lines and tissue types, compare drug response based on mutational status and correlate compound response.

The Cancer Cell Line Encyclopedia (CCLE) [48] provides another critical piece in cancer research, offering detailed genetic and molecular information on a wide range of cancer cell lines [49, 105, 106]. Building upon this, the DepMap portal [107] presents as a precious tool for functional genomics. DepMap utilizes the data from CCLE to identify essential genes for cancer cell survival, employing cutting-edge CRISPR technology. This integration allows researchers to perform in-depth analysis of CCLE data within the DepMap framework, enhancing our understanding of cancer dependencies and paving the way for new therapeutic approaches targeting these vulnerabilities in cancer cells.

The SCLC-CellMinerCDB tool at the National Cancer Institute (NCI) stands out for its integration of diverse databases, including GDSC, CCLE, UT Southwestern (UTSW) Medical Center [108] and NCI-SCLC [17, 109]. This integration not only consolidates a wealth of data but also facilitates advanced analysis capabilities. Researchers can seamlessly explore and compare data from multiple sources, encompassing diverse omics datasets such as gene mutation/copy-number data, expression data, epigenetics data (DNA methylation and enhancer signal, [97, 110]) and gene signature enrichment, which can be compared to each other or to drug response data.

The cBioPortal [111] for Cancer Genomics is an excellent example of the power of integrative data analysis. It provides researchers with a multifaceted view of molecular data sets alongside clinical attributes. This tool is particularly adept at uncovering biomarkers for SCLC and for cancer in general. With cBioPortal, we can interrogate and visualize mutation distribution in patient cohorts, identify co-expressing or anti-expressing genes, or even compare survival between patient groups based on mutational status of selected genes. In addition, processed data and clinical information can be easily obtained, helping researcher create custom analyses from a curated set.

The integration of data repositories and analytical tools is crucial in navigating the complex molecular landscape of SCLC, representing the forefront of precision oncology. The future of SCLC research and treatment depends on the continued fusion of data acquisition with analytical sophistication, which holds the key to unlocking new realms in cancer therapy.

## Developing non-invasive diagnostics

SCLC is histologically characterized as a malignant epithelial tumor composed of small cells that feature minimal cytoplasm, indistinct cell borders, finely granular nuclear chromatin, and either absent or barely noticeable nucleoli. The majority of SCLC cases, roughly 90%, fall into the category of typical SCLC, which exclusively comprises these small cells. The rest are identified as combined SCLC, where the tumor also includes elements of large cell carcinoma [112]. SCLC can be classified into two stages: limited disease (LD-SCLC), when it is limited to the hemithorax, where radiochemotherapy is effective; or extensive disease (ED-SCLC), where metastatic disease can be found outside of the hemithorax at diagnosis [113].

The diagnostic process for SCLC typically includes a physical examination, an assessment of the patient’s performance status, laboratory tests, and various imaging techniques. These imaging techniques often comprise contrast-enhanced CT scans of the chest and abdomen, brain imaging through MRI or CT, and potentially FDG PET/CT for cases of limited-stage disease [114]. Prior to initiating treatment, a definitive tissue diagnosis of SCLC is necessary. The choice of sampling method for diagnosis largely depends on the anatomical location of the tumor [115]. Depending on the tumor’s position in the chest, biopsies can be performed using bronchoscopy, mediastinoscopy, endobronchial ultrasound (EBUS), transthoracic needle aspiration, or thoracoscopy, if required. Obtaining biopsy samples of distant metastases is often recommended as it not only aids in diagnosing the tumor but also confirms the advanced stage of the disease [112].

Nonetheless, obtaining a tissue biopsy involves invasive methods and is not always feasible or repeatable. Moreover, the quality and quantity of the samples are frequently inadequate [116]. This underscores the necessity for investigating new diagnostic techniques.

**FIGURE 4:**
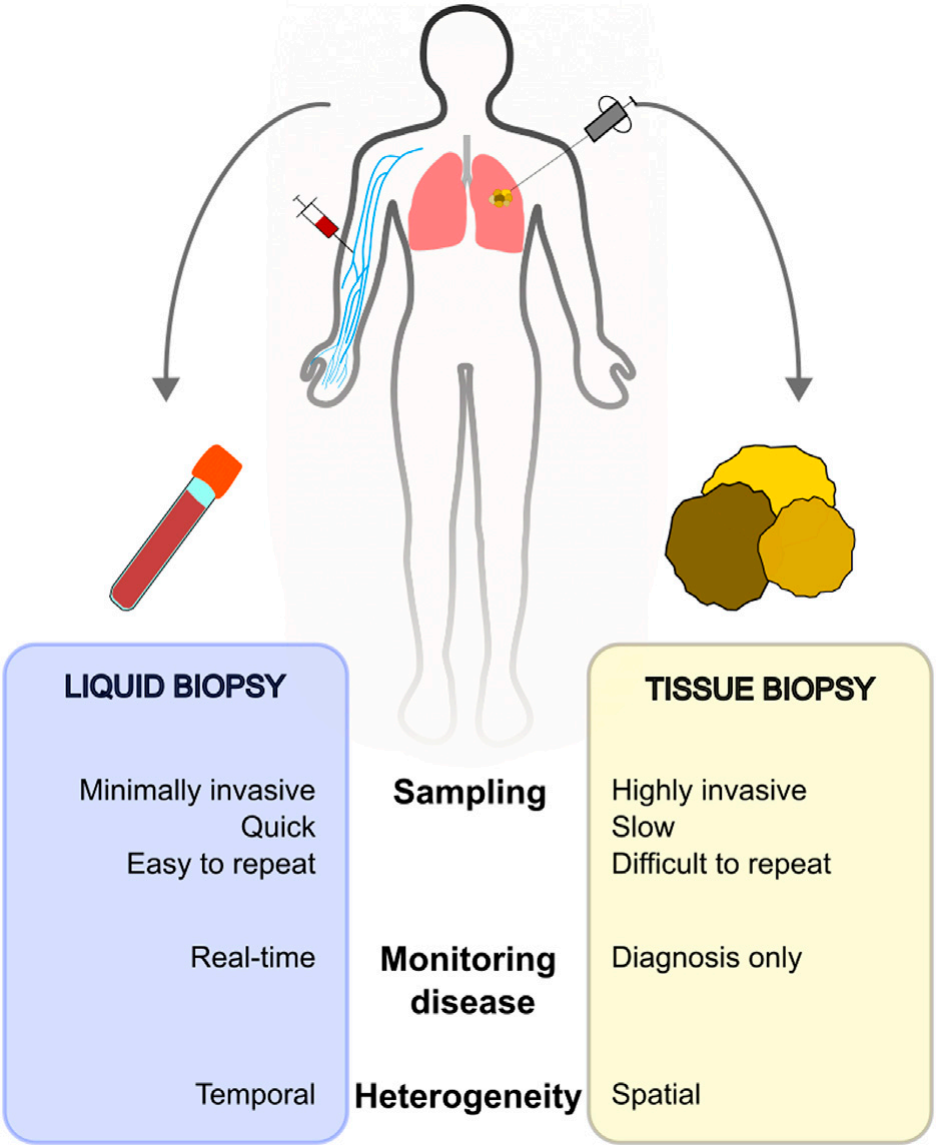
Comparison of liquid biopsy and tissue biopsy sampling.

Presently, new methods are emerging that address the limitations of traditional biopsies, such as liquid biopsy. Liquid biopsy involves analyzing biomarkers present in non-solid biological tissues, mainly blood. This technique offers significant benefits compared to conventional methods (Figure 4). The most extensively researched non-invasive cancer biomarkers include CTCs [117, 118], circulating tumor DNA (ctDNA) [119, 120], and circulating cell-free DNA (cfDNA) [96, 121, 122]. These circulating biomarkers are crucial for early cancer detection and can help determine the tissue of origin and prognosis. Additionally, they are useful in monitoring treatment responses, assessing potential resistance to therapies, and detecting minimal residual disease.

Although CTCs and ctDNAs often provide a more precise indication of tumor burden, the concentration of cfDNA still holds relevance in cancer management. Measuring total cfDNA concentration is more cost-effective than analyzing ctDNA or CTCs, which necessitate the use of expensive assays [123, 124]. While cfDNA can be increased in healthy patients for various reasons, ctDNA detection is more specific to tumors. Mutations identified in ctDNA samples are highly similar to those identified in the matched tumor tissues [125].

Due to the rapid growth and highly metastatic capacity of SCLC tumors, ctDNA levels can be valuable markers. Among others, TP53 and RB1 alterations play an important role in SCLC tumorigenesis, and can be used for monitoring of relapse through ctDNA sequencing [121, 125–127]. Fernandez-Cuesta et al. studied the possibility of detecting TP53 mutations from ctDNA. They were able to detect TP53 mutations in 35.7% of early-stage SCLC patients and 54.1% of late-stage SCLC patients [128]. Herbreteau et al. extracted circulating DNA from plasma and detected mutations in the TP53, RB1, NOTCH1, NOTCH2 and NOTCH3 genes using targeted next-generation sequencing [126]. Circulating tumor DNA was detectable if at least one somatic mutation was identified. Overall, mutations in TP53, RB1, and NOTCH1–3 genes were identified in 49 of 68 patients (70.6%), where the most frequently identified mutations affected TP53 (32/49; 65.3%) and RB1 (25/49; 51.0%) genes. Interestingly, almost a quarter of the patients harbored at least one mutation in one of the NOTCH genes (12/49; 24.5%), consistent with results seen in tumor samples [7].

In order to understand the subclonal architecture of SCLC, Nong et al. analyzed the cfDNA samples of 22 SCLC patients before and at different points in therapy using a panel of 430 genes [125]. All patients had a somatic mutation at baseline, the most common being the TP53 mutation, which was observed in 91% (20/22) of patients, and the RB1 mutation, which was observed in 64% (14/22) of patients. Overall, over 90% of patients had mutations in TP53, RB1, or both genes, and 27.3% had NOTCH1–3 mutations. In addition, plasma and tissue samples from eight patients were analyzed, showing a 94% concordance for mutations, indicating that cfDNA sequencing is a sensitive tool for detecting somatic mutations in SCLC patients. Despite the high concordance in the patient cohort, in one case none of the 26 mutations detected in tumor tissue were found the matched cfDNA sample. Also, two of the discordant cases became positive after increasing the sequencing depth. Importantly, in some patients a subset of mutations was detected exclusively in cfDNA, which may be a cause of tumor heterogeneity. Overall, a similar subclonal architecture was revealed between tissue and cfDNA, supporting the use of cfDNA to detect somatic mutations and study molecular heterogeneity in SCLC.

Serial plasma samples from 27 SCLC patients were analyzed by Almodovar et al., where disease-related mutations were detected in 85% of patients. TP53 and RB1 were the most frequently altered genes, and 10 additional genes (PTEN, NOTCH1–4, MYC, MYCL1, PIK3CA, KIT and BRAF) were detected in 52% of patients. In nine patients, cfDNA changes preceded radiological evidence of relapse [127]. Consistent with other studies, ctDNA monitoring has also been shown to identify disease recurrence prior to disease progression seen on imaging or in cases where imaging is equivocal [121, 122, 127]. Similar results were found in other studies, where cfDNA levels were found to be associated with disease outcome, as patients with high levels had a worse prognosis [129].

## Conclusion

The fight against SCLC has been a path filled with both obstacles and progress. The disease’s rapid spread, limited treatment choices, and the typically brief survival periods of patients have highlighted the urgent need for ongoing improvement and innovation in treatment methods and diagnostics. Our growing knowledge of SCLC is being fueled by the use of cell lines, patient-derived organoids, and mouse models, coupled with the rise of multi-omics studies and cutting-edge computational techniques. These help us better understand genetic and epigenetic changes that regulate SCLC, which may be exploited as potential therapeutic vulnerabilities. In addition, the field of diagnostics has undergone significant transformation. The limitations of traditional, more invasive biopsy methods and the scarcity of surgical specimens have given rise to advanced techniques such as liquid biopsies. These modern approaches, which analyze biomarkers like circulating tumor cells, circulating tumor DNA, and circulating cell free DNA, provide a less invasive and more dynamic perspective on the genetic makeup of the tumor and its response to treatments. As this journey progresses, each new breakthrough offers renewed hope and enhances our understanding of this complex and formidable disease.

## Footnotes

1
https://www.nccn.org/
